# The Catalytic Machinery of a Key Enzyme in Amino Acid Biosynthesis

**DOI:** 10.4061/2011/352538

**Published:** 2010-12-22

**Authors:** Ronald E. Viola, Christopher R. Faehnle, Julio Blanco, Roger A. Moore, Xuying Liu, Buenafe T. Arachea, Alexander G. Pavlovsky

**Affiliations:** ^1^Department of Chemistry, University of Toledo, Toledo, OH 43606, USA; ^2^Structural Biology Lab, Cold Spring Harbor Labs, Cold Spring Harbor, NY 11724, USA; ^3^Rocky Mountain Labs, National Institutes of Health, Hamilton, MT 59840, USA; ^4^Department of Pharmacology, Yale University, New Haven, CT 06520, USA

## Abstract

The aspartate pathway of amino acid biosynthesis is essential for all microbial life but is absent in mammals. Characterizing the enzyme-catalyzed reactions in this pathway can identify new protein targets for the development of antibiotics with unique modes of action. The enzyme aspartate **β**-semialdehyde dehydrogenase (ASADH) catalyzes an early branch point reaction in the aspartate pathway. Kinetic, mutagenic, and structural studies of ASADH from various microbial species have been used to elucidate mechanistic details and to identify essential amino acids involved in substrate binding, catalysis, and enzyme regulation. Important structural and functional differences have been found between ASADHs isolated from these bacterial and fungal organisms, opening the possibility for developing species-specific antimicrobial agents that target this family of enzymes.

## 1. Introduction

Enzyme-catalyzed reactions organized into sequential pathways are responsible for producing the molecules needed to sustain life. While many essential metabolic pathways are present in all known life forms, there are also significant differences between microbial and mammalian metabolism. Most microbial species can synthesize all of their important metabolic building block molecules, while mammals must acquire many of these from dietary sources. These metabolic differences provide a substantial number of potential protein targets to be examined for the development of selective antimicrobial agents. There is a growing need to identify effective new antibiotics that function against new targets to combat the expanding threat from pathogenic species that are becoming increasingly resistant to existing antibiotics [1, 2]. There are, however, fundamental problems that must be resolved for effective biocides to be developed against metabolic enzyme targets. Infectious microorganisms often use their host as a source of essential metabolites, thus bypassing inhibitors designed to block key steps in microbial metabolism. In many instances microorganisms have developed alternative routes for the production of important metabolites that can readily bypass inhibited enzyme reactions [3]. Essential enzymatic pathways can also be altered in response to an antibiotic threat. Although these microbial pathways may not exist in mammals, related enzymes with similar active site geometries or substrate binding motifs can potentially interact with even the most carefully designed inhibitors. The structure and mechanism of a bacterial metabolic enzyme must be thoroughly characterized before a potential drug target can be fully evaluated. Aspartate *β*-semialdehyde dehydrogenase (ASADH) is a key enzyme in an essential amino acid biosynthetic pathway that is not present in mammals. This enzyme has been thoroughly investigated and is now being examined as a target for the development of new antimicrobial agents.

## 2. The Aspartate Biosynthetic Pathway in Microbes

**Figure 1:**
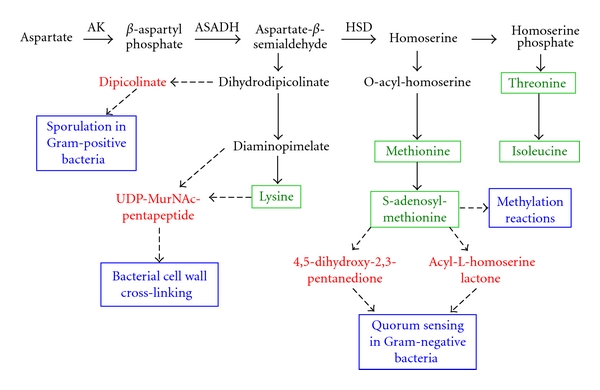
The aspartate biosynthetic pathway in microorganisms. The end product amino acids produced by this pathway are shown in green. Pathway-specific metabolites (shown in red) play crucial roles in microbial life cycle functions (shown in blue).

The aspartate biosynthetic pathway is present only in plants and microbes. The commitment step to this pathway is the phosphorylation of aspartic acid catalyzed by a family of aspartokinases (AK). The next enzyme in the pathway, ASADH, catalyzes the production of aspartate semialdehyde (ASA) that is located at a critical junction in this pathway. From this point one pathway branch leads to the production of lysine through the metabolite diaminopimelate, while the alternative route leads to the synthesis of methionine, threonine, and isoleucine with homoserine serving as their common intermediate (Figure 1). Thus, one quarter of the amino acids required for protein synthesis in all organisms are linked through and synthesized by this pathway [4]. The aspartate pathway is exquisitely regulated to control the total amino acid output as well as the relative levels of each amino acid. This regulatory scheme must also maintain the levels of several essential metabolic intermediates during periods of low protein synthesis. This is accomplished by coordinated regulation by the end product amino acids, both through feedback inhibition and by selective gene repression [5]. This regulatory scheme allows each end product to modulate the flux through the initial pathway steps, with branch point allosteric enzymes providing further control over the product levels. 

In addition to the essential amino acids produced in the aspartate pathway, several important metabolites are synthesized that play crucial roles in important developmental processes, such as cell wall biosynthesis, protective dormancy, and virulence factor production. For example, dihydrodipicolinate is a precursor of dipicolinate, the major component of bacterial spores [6], and diaminopimelate (DAP) is required for cross-linking of the peptidoglycan polymers [7] in bacterial cell wall synthesis (Figure 1). Another product of this pathway, *S*-adenosylmethionine, is an essential methyl group donor that also serves as a precursor for quorum sensing signaling molecules with critical roles in triggering virulence factors in infectious microorganisms [8, 9]. Homoserine lactone signals, for example, are essential for both virulence and biofilm development in the human pathogen *Acinetobacter baumannii* [10].

Because this pathway produces many essential compounds that are involved in a range of critical functions, disruptions to the aspartate pathway are fatal to those microorganisms. In particular, selective perturbations of the *asd* gene that encodes for ASADH are lethal to numerous infectious microorganisms. For example,* asd* mutants of *Salmonella typhimurium* develop an absolute growth requirement for diaminopimelate (DAP), a critical cell wall cross-linking component (Figure 1) in Gram-negative bacteria [11]. This mutated organism undergoes cell lysis when DAP is not supplied, and, since this metabolite is not produced in mammals it cannot be supplied by the host organism. A similar loss of viability is observed in *asd*-deficient *E. coli* strains. During amino acid starvation microorganisms often use specific transport systems to import exogenous amino acids available from the host environment [12]. However, *de novo* biosynthesis of lysine is essential for the survival of *Mycobacterium tuberculosis* during infection in mice, despite the presence of lysine in the host [13]. Even if an organism could mutate to improve lysine transport capacity in response to aspartate pathway inhibition, reversal of the decarboxylation that produces lysine from DAP is neither kinetically nor thermodynamically feasible. Both of these end products and several additional intermediates of this pathway are thus critical for microbial cell viability, both in culture and during host infection. Blockage of the aspartate pathway is fatal to microorganisms. Therefore the identification of effective inhibitors of key aspartate pathway enzymes should provide lead compounds for the development of new biocides. To achieve this aim we have focused on the functional and structural characterization of the microbial ASADH family of enzymes.

## 3. Sequence and Structural Comparisons among the Aspartate-***β***-Semialdehyde Dehydrogenases

**Scheme 1:**
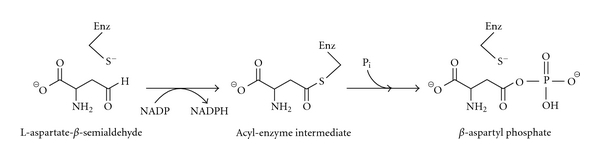
The conversion of aspartate *β*-semialdehyde to *β*-aspartyl phosphate catalyzed by aspartate *β*-semialdehyde dehydrogenase in the reverse (nonphysiological) reaction.

Aspartate-*β*-semialdehyde dehydrogenase (ASADH) catalyzes the second reaction in the aspartate pathway, the reductive dephosphorylation of *β*-aspartyl phosphate to aspartate-*β*-semialdehyde (ASA) (Scheme 1), at a critical branch point in this pathway. 

**Figure 2:**
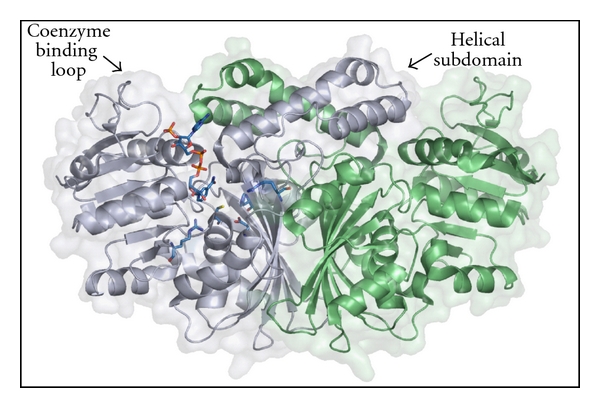
Ribbon drawing and surface rendering of the ASADH from *Escherichia coli *showing the overall structure. Each of the ASADH structures shares a conserved domain organization and exists as a functional homodimer. The largest structural differences between the different branches of this enzyme family manifest themselves in the coenzyme binding loop and in the helical subdomain bridging the dimerization interface. The bound coenzyme NADP and the active site amino acids are shown in one subunit as blue sticks.

The ASADHs from a variety of organisms encompass a considerable diversity of sequence homologies, ranging from less than 10% to as high as 95% sequence identity when compared to the *Escherichia coli* enzyme (*ec*ASADH). The ASADH enzymes in microorganisms can be divided into three branches consisting of the enzymes from Gram-negative bacteria, Gram-positive bacteria, and archaea/fungi. These branches were initially identified and partitioned through sequence alignments and now, with representative high resolution structures available from each branch, have been compared by structural alignments. The earliest structures of ASADHs are from enzymes that were isolated and purified from Gram-negative bacteria. These enzyme forms share significant sequence and structural homology and include structures of the ASADHs from *E. coli* [14, 15], *Vibrio cholerae* [16], and *Haemophilus influenzae* [17]. The overall structure of these ASADHs is a homodimer with an extensive contact surface between the subunits. Each monomer is composed of a carboxy-terminal domain primarily involved in hydrophobic intersubunit contacts, and a more hydrophilic amino-terminal domain that forms the active site and NADP binding site (Figure 2). 

The ASADH from *Streptococcus pneumoniae* (*sp*ASADH) is the first member of the Gram-positive bacterial branch that was structurally characterized [18]. *sp*ASADH is a good representative of the other Gram-positive bacterial ASADHs with greater than 40% sequence identity to these enzymes, while having less than 25% identity with any of the Gram-negative enzymes. Unexpectedly, this *sp*ASADH has lower sequence homology to the Gram-negative enzymes than to those of the archaeal/fungal branch, with sequence identities from this comparison ranging from 18 to 30%. The structure of the ASADH from the archaeal hyperthermophile *Methanococcus jannaschii* (*mj*ASADH) has a similar overall fold and domain arrangement as the Gram-negative enzyme forms [19] despite being less than 10% sequence identity. But the complete set of functionally important active site amino acids has been conserved in *mj*ASADH, suggesting an identical mechanism in the enzyme from this ancient organism despite its lower catalytic efficiency. The first structure of an ASADH from a fungal species, *Candida albicans* (*ca*ASADH), was recently determined, and it also possesses a similar overall fold, domain organization, and active site structure as the other members of this enzyme family [20]. This fungal enzyme has less than 30% sequence identity to any of the bacterial ASADHs and only 40% identity to its closest homologue. Alignment of the structures that have been determined from each branch of the ASADH family shows that both *sp*ASADH and *ca*ASADH are most similar to the archaeal ASADH from *M. jannaschii *and more distantly related to the Gram-negative enzymes. 

Despite the overall sequence and structural similarities, there are a number of insertions and deletions which serve to differentiate the three ASADH branches. Structural variations between these branches include a sequence of residues on the enzyme surface that contribute to the binding pocket for the coenzyme NADP. The* E. coli *ASADH is representative of the Gram-negative enzymes, with a highly flexible coenzyme binding loop in the absence of NADP that becomes ordered in response to NADP binding [15]. The archaeal *mj*ASADH has three conserved insertions totalling 30 residues when aligned against the Gram-negative bacterial *ec*ASADH [19], with each insertion located on the surface of the structure. A 13-residue insertion in *mj*ASADH leads to an alternative orientation of the coenzyme binding loop, differing from that in the Gram-negative enzymes by about 90°, causing it to drape over and occlude the coenzyme binding pocket [19]. In addition, the helical subdomain that comprises a significant fraction of the dimer interface (Figure 2) is absent in the archaeal *mj*ASADH. The Gram-positive *sp*ASADH has the same number of residues in this region as the Gram-negative bacterial enzymes, but two short *β*-strands replace the helix-turn-helix structure observed in the helical subdomain of *ec*ASADH (Figure 2). The fungal enzyme from *C. albicans* is also missing the helical subdomain [20] and contains most of the insertions and deletions observed in the archaeal enzyme. These structural changes suggest differences in how each branch of this enzyme family can carry out its catalytic role, even though each possesses an identical repertoire of highly conserved active site functional groups.

## 4. Role of Active Site Functional Groups

**Figure 3:**
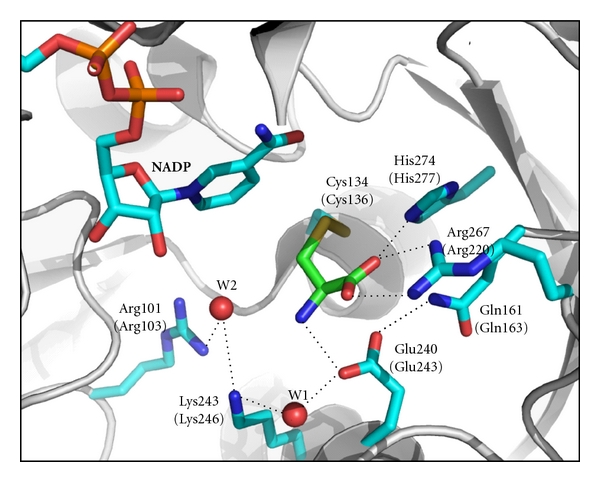
The active site structure of *Vibrio cholerae* ASADH with bound NADP and the covalent inactivator SMCS (shown in green). Cys134 is the active site nucleophile, and His274 is the acid-base catalyst. Glu240 and Arg267 are substrate binding groups, with Arg101 and Lys243 comprising part of the phosphate binding site that is occupied in this structure by a water molecule (W2). *H. influenzae* numbering is in parentheses (figure adapted from reference [16]).

In spite of the overall sequence diversity between the different branches of the ASADH family the identity of the core active site functional groups has been preserved throughout evolution (Figure 3). A set of active site mutants of ASADH from *H. influenzae* (*hi*ASADH) was examined kinetically and structurally with the goal of more precisely establishing the role for each functional group in substrate recognition and binding. A cysteine had been previously identified from biochemical studies as the likely active site nucleophile [21]. Replacement of a single sulfur atom with an oxygen in the C136S mutant virtually eliminates catalysis (Table 1), supporting the essential role of this residue as the catalytic nucleophile. Some decrease in catalytic activity would be expected because the hydroxyl group of serine is a weaker nucleophile than a cysteine sulfhydryl group. Only minor changes are observed in the active site structure of this mutant despite the nearly complete loss of catalytic activity. But a change in orientation of the introduced hydroxyl group is likely responsible for the very low activity of this mutant. The side chain hydroxyl group of the introduced serine rotates by about 90 degrees, with this new orientation stabilized by a hydrogen-bond to an adjacent backbone carbonyl group [22]. This reorientation not only moves this functional group out of the position needed to act as a nucleophile, but also competes with the involvement of this backbone carbonyl in intermediate stabilization and alters the position of the bound phosphate group.

Mammalian glyceraldehyde-3-phosphate dehydrogenase (GAPDH) catalyzes a similar reaction, an oxidative phosphorylation, to the reverse reaction catalyzed by ASADH and appears to do so by the same mechanism [23]. Before the first structure of ASADH was available, sequence alignments to the mechanistically related enzyme (GAPDH) family were used to identify and then test the roles of possible active site functional groups. Both enzymes were proposed to contain a cysteine-histidine catalytic dyad; however the corresponding position of the catalytic histidine in GAPDH is occupied by a conserved glutamine in ASADH [24]. Replacement of this functional amino acid causes a loss in catalytic activity that is not fully recovered even when a histidine is introduced at this position in ASADH. This mystery was solved when the first ASADH structure revealed that the essential histidine residue actually comes from a completely different position in the primary sequence, with a loop containing H277 folded into the enzyme active site [14]. The H277N mutant of *hi*ASADH has significantly impaired activity (Table 1), highlighting the key role for this residue. However a ternary complex structure with NADP and the active site-directed inactivator, S-methyl-L-cysteine sulfoxide (SMCS), showed continuous density extending from the Cys136 nucleophile [22]. This structure is consistent with the covalent attachment of SMCS to the active site nucleophile. The position of this bound inactivator is shifted in the H277N mutant relative to its position when bound in the wild-type enzyme [15]. This reorientation moves this compound further from the bound phosphate that must attack the carbonyl carbon of the acyl-enzyme intermediate to generate *β*-aspartyl phosphate in the reverse reaction (Scheme 1). 

So in summary for these catalytic mutants, the C136S mutant still allows substrate binding but formation of the covalently bound intermediate is slowed by a less efficient and misoriented nucleophile. In contrast, the H277N mutant appears to form the initial tetrahedral intermediate efficiently, but its subsequent breakdown is hindered by a shift away from the bound phosphate nucleophile. In each case, small perturbations in the positioning of essential catalytic groups or reactive intermediates are shown to have dramatic effects on enzyme catalysis.

Additional mutant structures of *hi*ASADH have been determined, with each substitution replacing a putative substrate binding group in order to assess their function. Each of these mutants displayed significantly impaired catalytic activity, ranging from *∼*10% to less than 0.1% that of the wild-type enzyme [25]. However, the structural basis for the diminished activities is different for each mutation (Table 1). A conserved arginine (Arg270) in the ASADH family aligns with a conserved Arg231 in the GAPDH family, a residue that had previously been assigned a role in binding the substrate phosphate group [26]. Based on kinetic studies this arginine residue was proposed to have a comparable role in *hi*ASADH, namely, binding the carboxyl group of the substrate ASA [24]. The structures of substrate complexes of the wild-type enzyme [17] and of the inactivator SMCS covalently bound to *Vibrio cholerae* ASADH (*vc*ASADH) [16] support this assignment by clearly establishing the presence of a bidentate interaction between Arg270 and the carboxylic group of the substrate ASA. 

Arginine270 was replaced by lysine in *hi*ASADH to assess its relative importance in substrate binding by disrupting the bidentate interaction that is formed with ASA. Maintaining this interaction between the introduced lysyl amino group and the ASA carboxyl group will require a shift in the orientation of both the lysyl side chain and the resulting tetrahedral intermediate relative to their positions in the wild-type enzyme. The dramatic loss of activity in R270K can be explained by a shift of the intermediate away from the position that is necessary to form and maintain a productive interaction between its carboxyl group and the altered substrate binding residue during the catalytic cycle. A neutral side chain introduced at this position in *ec*ASADH (R267L) cannot form any binding interaction with the substrate carboxyl group, leading to a 30-fold increase in the *K*_m_ for ASA [24] while also permitting greater flexibility for positioning of the covalent intermediate. This flexibility increases the likelihood that the intermediate can adopt a catalytically viable conformation relative to that imposed in the *hi*ASADH R270K mutant, and this conformational flexibility is manifest in the 100-fold greater activity seen in the R267L *ec*ASADH enzyme form (Table 1). 

Glutamate243 provides a side chain carboxyl group in the active site of ASADH whose role in the catalytic cycle had not been definitively established. This group is highly conserved among ASADHs from different organisms, and structural studies have shown that it is in position to potentially interact with the amino group of the tetrahedral intermediate in the *hi*ASADH complex [17]. An E243D mutant was produced to assess the possible role of this residue in substrate and intermediate binding. Kinetic studies of E243D failed to show the expected detrimental effect on substrate interactions, with the *K*_m_ for ASA unchanged from that of the wild-type enzyme. Instead the catalytic efficiency of this mutant is significantly compromised, reducing the *k*_cat_ value to about 1% of that of wild-type *hi*ASADH (Table 1). In this case the position of the bound intermediate (and presumably that of the bound substrate) shifts towards the shorter side chain of the introduced aspartate at this position. This shift allows this mutant to maintain substrate binding affinity, but compromises the positioning between the intermediate and the bound cofactor thereby leading to impaired catalytic efficiency.

Each of these mutations was prepared with the aim of removing and testing critical substrate binding groups. However the structural rearrangements that occur as a consequence of these replacements manifest themselves not in a loss in substrate binding affinity, but in a loss of catalytic activity. Structural characterization of these mutants shows that only subtle changes in key active site residues, such as rotation of a side chain to form a new hydrogen-bond or a shift in position relative to a catalytic intermediate, are sufficient to adversely affect catalysis.

**Figure 4:**
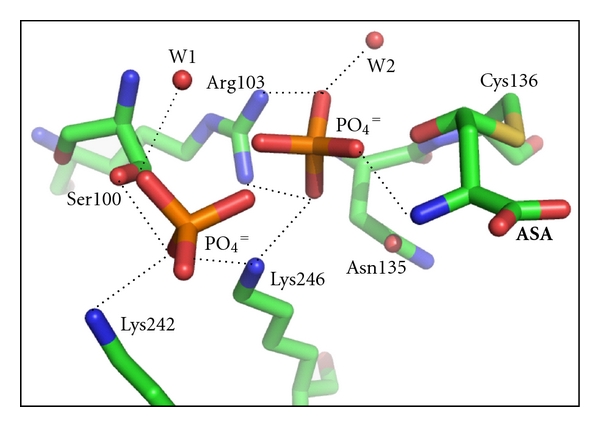
The oxyanion binding site in *H. influenzae* ASADH. Each of the oxyanion substrates and inhibitors interacts with the same protein ligands, Arg103 and Lys246, and is bound within attacking distance of the covalent acyl-enzyme intermediate. In the substrate structures with either phosphate or arsenate a second oxyanion molecule is bound to Ser100, Lys242, and Lys246 (figure adapted from reference [28]).

The phosphate-binding site of ASADH is capable of accommodating different tetrahedral oxyanion analogues [27], leading to different functional consequences. Both oxyanion substrates and oxyanion inhibitors bind in the same position by using the same set of ligands (Figure 4), raising the question of what distinguishes a substrate from an inhibitor? In the apoenzyme the side chain hydroxyl of Thr137 forms a hydrogen-bond with Asn135, and this pairing prevents any interactions with the active site Glu243. Upon binding of either oxyanion substrate, phosphate or arsenate, Thr137 switches hydrogen-bonding partners through a 60° rotation that disrupts its interaction with Asn135. This new conformation moves the threonine hydroxyl group into position to interact with and orient Glu243, an important substrate binding group. Glu243 remains in this substrate binding position in the arsenate structure, and this hydrogen-bond with the Thr137 hydroxyl group persists even in the absence of bound ASA. So, the presence of an oxyanion substrate in the active site helps to position Glu243 to interact with the substrate. However, in the presence of the inhibitor periodate this threonine does not change position and switch partners. In this inhibitor-bound structure Thr137 remains hydrogen-bonded to Asn135, which is now oriented away from Glu243 and cannot stabilize the position of this side chain. This single change in hydrogen-bonding partners is apparently sufficient to interfere with the binding of ASA. Thus, subtle shifts in the position of side chains, even those not directly involved in substrate binding or catalysis, can lead to a sequence of events that result in loss of function for a finely tuned enzyme catalyst.

## 5. Differences in Coenzyme Binding and Specificity

**Figure 5:**
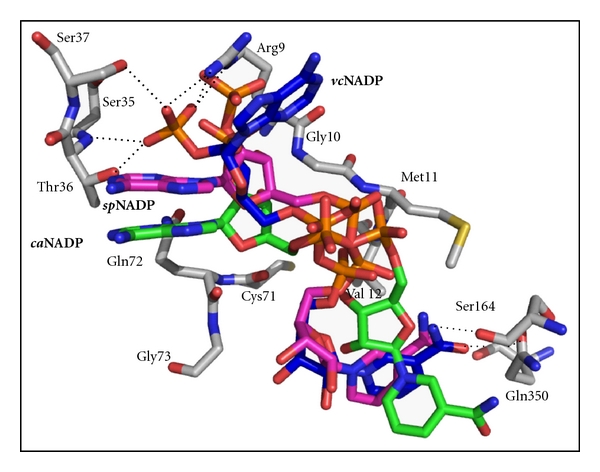
Differences in coenzyme binding in ASADHs. An overlay of the NADP binding orientations in *V. cholerae* ASADH (blue), *S. pneumonia* ASADH (red), and *C. albicans* ASADH (green). The center of the adenine ring in the Gram-positive and fungal enzymes has shifted by about 8.5 Å with respect to its position in *vc*ASADH, and the position of the exocyclic amine on C6 has moved by nearly 14 Å (figure adapted from reference [20]).

The active site functional groups of ASADH are already poised to accommodate amino acid substrate binding in the apoenzyme. However, the binding of NADP is required to induce a domain closure that sets up the active site for catalysis. NADP binding and the coupled domain closure are driven by numerous interactions between the enzyme and the molecular features that are distributed throughout the coenzyme. In *vc*ASADH, Arg9 is repositioned during NADP binding to form an electrostatic interaction with the 2′-phosphate, with additional hydrogen-bonds to phosphate from Thr36 and Ser37 (Figure 5). A consensus sequence, **SG**x**G**, present in each branch of the ASADH family interacts *via* backbone carbonyl hydrogen-bonds to the amide nitrogen of the nicotinamide, while a conserved glutamine (Gln350) in the bacterial enzymes and a corresponding asparagine in the archaeal enzyme are in position to hydrogen-bond to the amide oxygen (Figure 5). 

Binding interactions to the adenine ring of NADP are less conserved between the Gram-negative and Gram-positive bacterial enzymes, leading to some drastic differences in coenzyme binding. The adenine base in *vc*ASADH is oriented by a cation-*π* interaction with Arg9 (Figure 5) in the consensus **G**xx**G**xx**G** sequence which is part of the Rossmann nucleotide fold [29]. A surface loop spanning from Leu189 to Ser195 closes around NADP in these Gram-negative enzymes, with the exocyclic N6 of the adenine base forming a hydrogen-bond with the backbone carbonyl of a proline (Pro192) located on the helical subdomain from the opposite subunit of the dimer (Figure 2). This interaction plays a critical role in the change from an open to a closed enzyme conformation upon coenzyme binding [16]. The overall domain movements encountered in the transition from the apoenzyme to the NADP complex in Gram-positive *sp*ASADH are similar to those observed for *vc*ASADH [15]. Rotation of the N-terminal domain toward the active site in response to NADP binding appears to be a universal mechanism utilized throughout the ASADH family. However, none of the interactions that drive domain closure in both *ec*ASADH and *vc*ASADH are observed in *sp*ASADH. Thus, facilitation of the commonly observed domain closure in *sp*ASADH must be driven by a different set of interactions than those that drive the same closure in the Gram-negative ASADHs. 

A completely new binding pocket for the adenine base is found in *sp*ASADH, along with an altered 2′-phosphate binding site, from that previously observed in the Gram-negative enzymes. The adenine binding pocket in *sp*ASADH is forged between an *α*-helix and the coenzyme binding loop of the N-terminal domain. Interactions between the adenine ring and the side chains of Thr76 and Ser37, along with a cation-*π* interaction with Arg39, serve to anchor the adenine ring in this pocket [18]. These new interactions cause the adenine ring to adopt an altered position in *sp*ASADH formed by rotation around the bonds linking the nicotinamide and adenine ribose with the bridging diphosphates. As a consequence the center of the adenine ring is shifted by about 8.5 Å with respect to its position in NADP bound in *vc*ASADH, and the position of the exocyclic amine on C6 that formerly interacted with Pro192 from the adjacent subunit has been displaced by nearly 14 Å (Figure 5). However, the 2′-phosphate of NADP bound in the *sp*ASADH structure moves less than 5 Å relative to its position in *vc*ASADH, and the phosphate binding groups move to accommodate this shift.

 Comparison of the coenzyme binding site between archaeal *mj*ASADH and the bacterial enzymes also shows some conserved similarities. In bacterial ASADHs the backbone amino groups from a methionine and valine are hydrogen-bond donors to the pyrophosphate of NADP (Figure 5), and a change from methionine to serine in the *mj*ASADH structure does not alter these pyrophosphate interactions. A similar reorientation of the adenine ring to that observed in *sp*ASADH is seen in the binding of NADP to the ASADH from *M. jannaschii *[19]. An extended surface loop in *mj*ASADH (from residues 40 to 77) forms the binding pocket for this conformation of the bound NADP. Specific contacts between the adenine ring and the enzyme include two hydrophobic interactions to Leu91 and Leu95 that are within 4.0 Å of the plane of the adenine ring and a cation-*π* interaction with Arg39. Similar interactions and a closely related coenzyme orientation are also found in the binding of the adenine ring in the fungal enzyme structure (*ca*ASADH) [20] (Figure 5). 

These changes in coenzyme conformation and in the nature of the interactions with the 2′-phosphate of NADP in the *mj*ASADH structure suggest the possibility of altered coenzyme specificity relative to that seen for the well-studied Gram-negative bacterial enzymes. Preference for NADP *versus* NAD binding in enzymes is mediated through specific interactions with either the 2′-phosphate of NADP or the 2′-hydroxyl of NAD. The ASADHs from *E. coli*, *V. cholera*e, and *H. influenzae* are each highly specific for NADP. The structure of the NADP complex in *vc*ASADH shows that the 2′-phosphate group is bound by the same arginine that stacks against the adenine base, along with three hydrogen bonds that are formed with two serines and a threonine. This arginine in *vc*ASADH is not conserved in *mj*ASADH but instead is replaced by a threonine that is still in the correct position to form a potential hydrogen-bond with the 2′-phosphate of NADP but, unlike in *vc*ASADH, is not capable of binding to the adenine base. 

The possibility of expanded coenzyme specificity for the archaeal enzyme was examined kinetically by testing both NADP and NAD as substrates near the physiological temperatures for this thermophilic organism. *mj*ASADH is found to have comparable catalytic rates with either NAD or NADP when examined at 70^*º*^C [19]. However, this enzyme still shows a preference for NADP over NAD as its coenzyme, with a Michaelis constant that is 130-fold lower for NADP (*K*_NADP_ = 13 *μ*M) compared to that for NAD (*K*_NAD_ = 1700 *μ*M). It appears that the relaxed specificity for NADP in *mj*ASADH is primarily due to absence of this critical arginine residue, since the positively charged guanidinium group of this arginine interacts directly with the negatively charged 2′-phosphate of NADP. An additional explanation for this altered coenzyme specificity comes from the structure of the NADP complex in which the adenine and ribose end of the NADP is bound in a completely different binding pocket in archaeal *mj*ASADH compared to the Gram-negative bacterial enzyme family.

## 6. The Presence of an Intersubunit Communication Channel

The subunit interface in the homodimer for both the bacterial and archaeal ASADHs is composed primarily of hydrophobic *β*-sheets. The Gram-negative bacterial ASADHs have the largest dimer interface, formed by a conserved hydrophobic *β*-sheet and complemented by the helical subdomain to create over 3400 Å^2^ of buried surface area (Figure 2). This subdomain is also present in the Gram-positive bacterial *sp*ASADH, but a 16-residue deletion results in a single *α*-helix followed by an unstructured loop forming the top portion of the dimer interface with about 2600 Å^2^ of total buried surface area. A 48-amino acid deletion in *mj*ASADH results in the complete removal of the helical subdomain that makes a significant contribution to the dimer interface in *ec*ASADH. As a consequence the thermophilic *mj*ASADH structure has a much smaller dimer interface of about 2000 Å^2^. The enzyme from *C. albicans* (*ca*ASADH) is also missing the helical subdomain, with the 44 amino acids that constitute this helix-turn-helix motif in the Gram-negative enzyme forms replaced by an unstructured 3 amino acid loop [20]. As a consequence this fungal enzyme has the smallest dimer interface (*∼*1800 Å^2^) and the lowest percentage of buried surface area among the structurally characterized ASADHs. Interestingly, there is good correlation between the dimer interface area and enzymatic activity, with the enzyme forms possessing the highest buried dimer surface having the highest catalytic activity [30]. This correlation suggests that intersubunit communication in the ASADH dimer is critical to promote highly efficient catalysis.

**Figure 6:**
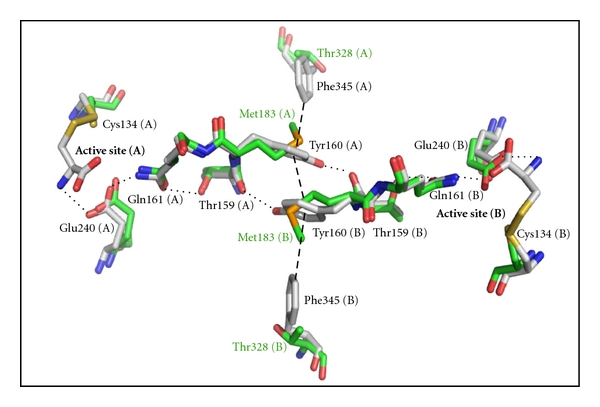
Proposed intersubunit communication channel in bacterial ASADHs. The subunit interface of *vc*ASADH (gray) is bridged by a hydrogen-bonding network (dotted lines) connecting the active sites of each subunit and stabilized by *π*-stacking interactions (dashed lines). In the subunit interface of *mj*ASADH (green) replacement of Tyr160 with Met183 interrupts the hydrogen-bonding network, and replacement of Phe345 with Thr328 disrupts the *π*-stacking stabilization.

A network of hydrogen bonds has been identified across the dimerization interface that is proposed to allow active site to active site communication in the functional ASADH dimer from the bacterial enzyme branch [16]. These structural observations support the earlier kinetic experiments that had suggested an alternating site reactivity model for ASADH catalysis [31]. The active site Glu240 in subunit A of *vc*ASADH is positioned through a hydrogen-bond to Gln161 (Figure 3). Gln161 also forms a hydrogen-bond with the backbone amide of Thr159, which in turn is hydrogen-bonded through its side chain hydroxyl group to the hydroxyl group of Tyr160 across the dimer interface in subunit B (Figure 6). A complementary series of interactions links the active site Glu240 in subunit B to Tyr160 in subunit A, such that each active site is linked to an amino acid in the adjacent subunit through a network of three hydrogen-bonds. The orientation of the Tyr160 residues at the domain interface is crucial to maintaining this network. The tyrosine side chain position is stabilized by *π*-stacking to the tyrosine from the other subunit and also by a perpendicular *π*-stacking interaction with Phe345 in the same subunit (Figure 6).

The structure of *mj*ASADH does not allow the intersubunit communication route that is seen in the bacterial enzymes. Two methionines are present within the dimerization interface region of *mj*ASADH that contribute to the hydrophobic subunit interactions, but replace the conserved tyrosines in each subunit that forms the heart of the hydrogen-bonding network bridging between the two active sites (Figure 6). The phenylalanines that stabilize these tyrosines through *π*-stacking have also been replaced in the archaeal ASADH branch with a conserved threonine (Figure 6). These amino acids that are crucial for intersubunit communication have also been replaced in the *ca*ASADH enzyme. The base-stacked tyrosine is substituted by either leucine or methionine at this position in all fungal ASADHs, while the stabilizing phenylalanine is now a conserved valine in this branch of the enzyme family [20]. These amino acid replacements disrupt the intersubunit hydrogen-bonding network observed in Gram-negative ASADHs, leading to the loss of this communication channel. The alternating sites reactivity observed in the bacterial forms of ASADH is likely absent in both the archaeal and fungal enzymes and could explain the very low catalytic activity in these enzyme forms despite the presence of an identical constellation of active site functional groups throughout the ASADH enzyme family.

## 7. Catalytic Mechanism and Covalent Intermediates

**Figure 7:**
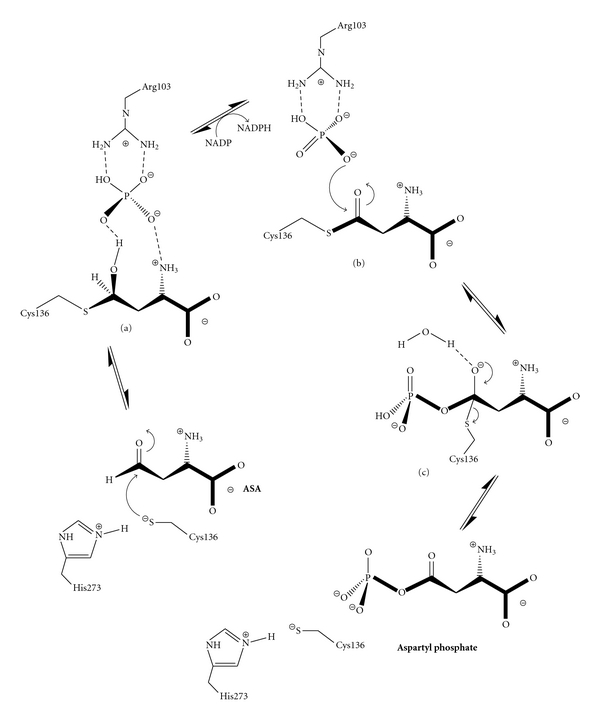
Catalytic mechanism of the reversible aspartate *β*-semialdehyde dehydrogenase-catalyzed conversion of ASA to aspartyl phosphate. (a) Tetrahedral intermediate derived from nucleophilic attack on ASA. (b) Acyl-enzyme intermediate produced by hydride transfer to NADP. (c) Proposed tetrahedral intermediate obtained from phosphate attack on the acyl-enzyme.

The proposed catalytic mechanism of ASADH in the reverse (nonphysiological) direction involves the initial attack of the active site cysteine nucleophile (Cys136) on the carbonyl carbon of the substrate ASA. The enzyme-bound tetrahedral intermediate produced by this reaction (Figure 7, structure a) is set up to transfer a hydride ion to the NADP coenzyme bound at an adjacent site, leading to an acyl-enzyme intermediate (Figure 7, structure b). Attack of bound phosphate at the carbonyl carbon of this intermediate produces a second enzyme-bound tetrahedral intermediate (Figure 7, structure c). Collapse of this intermediate with expulsion of the enzyme thiolate group yields the *β*-aspartyl phosphate product and leaves the enzyme ready to bind another molecule of ASA and repeat the catalytic cycle.

The chance to characterize a true intermediate in an enzyme-catalyzed reaction is a rare opportunity to bridge a mechanistic gap that is generally only hypothesized between the substrate and the product complexes, or extrapolated from intermediate-analogue structures. For the ASADHs this feat has been accomplished twice, capturing and structurally characterizing two different reactive intermediates in the catalytic cycle of this enzyme and lending structural support to this proposed mechanism.

**Figure 8:**
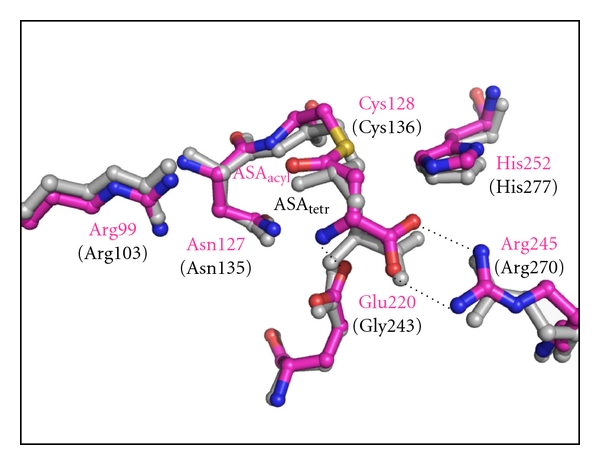
Overlay of the tetrahedral intermediate (ASA_tetr_, gray bonds and black labels) produced in *hi*ASADH and the acyl-enzyme intermediate (ASA_acyl_, magenta bonds and labels) obtained in *sp*ASADH. The change in geometry of the acyl carbon of this bound intermediate is tracked by shifts in the substrate binding groups, Asn127, Glu220, and Arg245. *H. influenzae* numbering is in parentheses.

A ternary complex produced by diffusing the substrates ASA and phosphate into crystals of *hi*ASADH was examined with the aim of determining the role of different active site groups in the catalytic mechanism. However, what was observed was not the structure of the enzyme-substrate complex, but instead the structure of the actual tetrahedral intermediate in the catalytic cycle of ASADH (structure a in Figure 7). The bound aspartyl intermediate was modeled into the continuous density emanating from the side chain of the cysteine nucleophile [17], and this structure clearly shows the tetrahedral geometry around the carbon that is covalently attached to the active site cysteine thiolate group (Figure 8). Subsequent hydride transfer from this tetrahedral intermediate to NADP would lead to formation of the acyl-enzyme intermediate. However, exclusion of the coenzyme from the crystallization conditions eliminates the hydride transfer step that is required for the reaction to proceed, thus stopping the catalytic cycle at this stage and allowing the determination of this important structure. 

The structure of the acyl-enzyme intermediate (structure b in Figure 7) was also determined by running the reaction in the nonphysiological direction. Soaking the *sp*ASADH-NADP complex crystals with the substrate ASA allows the catalytic cycle to proceed up to formation of the acyl-enzyme intermediate [18]. Exclusion of phosphate eliminates the nucleophile required to complete the catalytic cycle. Kinetic studies had previously shown that the acyl-enzyme can be quite stable in the absence of phosphate [32]. The refined structures show the key differences between the tetrahedral intermediate and the acyl-enzyme intermediate. The position of the substrate binding groups that interact with these intermediates shift only slightly between these two structures, with the largest movements caused by the conversion from a tetrahedral carbon geometry in the previous intermediate structure to a planar carbon geometry in the acyl-enzyme (Figure 8).

## 8. Concluding Remarks

Through a combination of kinetic, mutagenic, and structural studies the detailed catalytic mechanism of the aspartate *β*-semialdehyde dehydrogenases has been defined, and the essential functional groups that play a role in substrate binding, catalysis, and regulation have each been identified. Subtle changes in any of these critical functional groups are sufficient to alter the finely tuned catalytic machinery of this enzyme, leading to enzyme forms with significantly impaired activity. Differences have been observed between the members of this enzyme family, isolated from bacterial, archaeal, and fungal species, regarding their catalytic efficiency, their coenzyme selectivity, and their mode of catalysis. This extensive body of work on the different members of the ASADH family now allows us to evaluate this enzyme as a drug target. Compounds are now being screened [33] that can potentially recognize these structural and functional differences to produce initial inhibitors that can be developed into species-specific antimicrobials against this key enzyme in an essential metabolic pathway.

## Figures and Tables

**Table 1:** Kinetic and structural consequences of ASA dehydrogenase active site mutants.

Active site group^a^	Proposed function	Mutant	Catalytic activity^b^	Structural effect of the mutation	PDB code
Cys136 (Cys134)	Active site nucleophile	C136S	<0.01%	Rotation of S136 shifts the amino group of ASA and moves the bound phosphate by 1 Å	1PQP

His277 (His274)	Acid-base catalyst	H277N	1.0%	Breakdown of the initial tetrahedral intermediate is slowed by a shift in the phosphate position	1PQU

Arg270 (Arg267)	Binds the substrate carboxyl group	R270K R267L	0.1% 9.5%	The introduced lysine moves about 2 Å away from the original arginine position	1PS8

Glu243 (Glu240)	Binds the substrate amino group	E243D	1.2%	The bound intermediate shifts position by about 0.5 Å towards the shorter side chain of D243	1Q2X

Lys246	Helps orient bound phosphate	K246R	3.3%	The introduced arginine rotates by about 90° to form new interactions with S99 and K242	1PU2

Arg103	Binds phosphate	R103K R103L	0.4% 0.07%	Displaces the active site loop (N135 to S139) which shifts C136 away from H277	1PR3 1OZA

^a^ *H. influenzae* sequence numbering, with the numbers in parenthesis referring to the *V. cholerae* sequence

^b^ *k*_cat_ determined by varying ASA at fixed NADP levels and expressed as a percent of wild type enzyme activity

## Abbreviations

AK: Aspartokinases
ASA: Aspartate *β*-semialdehyde
ASADH: Aspartate *β*-semialdehyde dehydrogenase
DAP: Diaminopimelate
GAPDH: Glyceraldehyde-3-phosphate dehydrogenase
SMCS: S-methyl-L-cysteine sulfoxide
*ca:*: *Candida albicans*
*ec:*: *Escherichia coli*
*hi:*: *Haemophilus influenzae*
*mj:*: *Methanococcus jannaschii*
*sp:*: *Streptococcus pneumoniae*
*vc:*: *Vibrio cholerae.*
